# Effect of the implementation of a selective digestive decontamination protocol in an intensive care unit

**DOI:** 10.2478/jccm-2025-0025

**Published:** 2025-10-31

**Authors:** Rosario Fernández-Fernández, Eugenia Yuste-Ossorio, Natalia Chueca-García, Purificación Fernández-Morales, Rocio Morón-Romero, Manuel Colmenero

**Affiliations:** Hospital Universitario San Cecilio, Granada, Spain; Instituto de Investigación Biosanitaria, Granada, Spain

**Keywords:** selective digestive decontamination, critical care, nosocomial infection, colonization, multi-drug resistance organisms

## Abstract

**Introduction:**

The use of selective digestive decontamination (SDD) in critically ill patients remains controversial. The impact of antimicrobial resistance varies according to multiple factors attributed to the type of patient and the characteristics of intensive care units (ICU).

**Aim of the study:**

to describe the effect of the implementation of a selective digestive decontamination protocol on the incidence of nosocomial infections and colonization of multidrug-resistant organisms (MDRO) in an intensive care unit.

**Materials and methods:**

Prospective observational study in a general ICU of a University Hospital. All patients admitted for 2 years (divided into 1-year periods) before and after the implementation of the SDD were included. This intervention was performed in all patients who received invasive mechanical ventilation in the second period. Incidence density rates were determined for all nosocomial infections (per days of stay) and device-associated infections (per days of use), and risk ratio (RR) were calculated with 95% confidence intervals. Microbiological surveillance of the colonization status of patients was performed on admission and on a weekly basis. A univariate analysis was performed for comparison between groups. A p<0.05 was considered significant.

**Results:**

A total of 1532 patients were included in the pre-intervention period (pre-SDD) and 1734 in the post-intervention period (post-SDD). The incidence of all infections decreased [9.21 vs 6.54 per days of stay; RR: 0,71 (0,428 – 1,172), p=0,16], although not significantly. Both catheter-related bacteremias and all catheter-related bacteremias together (primary and secondary) were significantly reduced [4.49 vs 0.71 per 1000 days of use; RR: 0,157 (0,017 – 0,723), p=0,006]. The colonization rates by MDRO also decreased (3.26% vs 2.36%), but not significantly.

**Conclusions:**

Implementation of SDD significantly decreased the number of catheter-related bacteraemias, without an increase in MDRO colonization.

## Introduction

Infections caused by multidrug-resistant organisms (MDROs) remain a significant cause of morbidity and mortality in critically ill patients. ICUs are high-risk environments because of the vulnerability of patients, the use of invasive devices, and the frequent administration of broad-spectrum antibiotics [1]. Multiple preventive measures have been implemented and tested for the prevention of different device-associated infections in these patients [2]. Many of them have been grouped in strategies called bundles of measures, which have shown very positive results [3]. In Spain, the Spanish Society of Intensive Care Medicine and Coronary Units (SEMICYUC) and the Ministry of Health have designed and implemented, as part of the patient safety strategy, the ZERO projects [4].

The introduction of non-absorbable antibiotics to the digestive tract (selective oropharyngeal decontamination or SOD) combined with a short course of intravenous antibiotics (selective digestive decontamination or SDD) aims to reduce the burden of potentially pathogenic microorganism (PPM) by preventing infection [5]. This approach is based on the interruption of the mechanisms involved in the generation of ventilator-associated pneumonia. First, the upper digestive tract is colonized by these PPMs. Then, macro- or micro-inhalations that occur when there is an artificial airway (tube or tracheostomy cannula) favoring contamination of the tracheobronchial tree.

Although it is one of the most widely studied nosocomial infection prevention practices for critically ill patients, its use remains controversial. The results regarding the reduction of infections and mortality remain questionable. In the recently published Selective Decontamination of the Digestive Tract in the Intensive Care Unit (SuDDICU) trial, hospital mortality was not significantly different between patients with and without SDD [6]. However, when combined in a Bayesian metaanalysis, there was a 99.3% posterior probability that SDD was associated with reduced hospital mortality compared to standard care [7]. A Cochrane systematic review and meta-analysis [8] was recently published to assess the effect of these reviews on mortality and respiratory infections in patients receiving mechanical ventilation more than 48 hours. A total of 41 studies with around 11.000 patients were analyzed, and it was concluded that SDD reduces general mortality and respiratory infections and that SOD reduces respiratory infections, but not mortality.

Resistance induced by antibiotic use is classified according to the agent and number of antibiotic classes. Most commonly, if resistance is acquired during SDD, it is to one of the oral antibiotic groups (aminoglycosides) or those administered intravenously (cephalosporins). Much less frequent is non-susceptibility to three or more classes, which is known as multi-resistance. Although the risk of using SDD is associated with the appearance of resistance, multiple studies with different epidemiological designs and large sample sizes have shown that routine use of SDD is not associated with an increase in antibiotic resistance [9,10]. Even in long-term follow-up studies beyond the limits of the ICU, the absence of complications from its use has also been demonstrated. Despite this, the level of implementation in European ICUs remains poor because of concerns that many physicians still have about their favorable benefit-risk ratio. Schouten et al. advocated in a recent editorial: “As a starting point, an international consensus guideline/position paper on SDD is essential” [11].

The effectiveness of SDD can be affected by several factors, such as differences in ICU settings, variations in the implementation of SDD protocols, and the varying baseline prevalence of resistant organisms across institutions [12]. Depending on the incidence rates of nosocomial infections with RDOs, ICUs can be classified into those with a low or high rate (> 10 %), with the latter being those that attend a majority of patients with morbidity, immunosuppression, institutionalization, use of previous antibiotic therapy, complicated major surgeries, major burns, multiple infections, use of invasive devices, and long ICU stays. The usefulness of SDD was demonstrated in this type of ICU. Therefore, there is still a need to investigate its impact on different patient subgroups and ICU types. The aim of the present study was to analyze the effect of the introduction of a SDD program in a multipurpose ICU on both the incidence of nosocomial infections and colonization by multidrug-resistant organisms.

## Patients and Methods

This is a prospective quasi-experimental study with a before-after analysis of the intervention. All patients admitted to the ICU of a 22-bed University Hospital for two years (June 2021 to July 2023) were included. Patients who died within 24 hours of ICU admission were excluded. The cohort was divided into two periods of one year each, with the intervention being started halfway through. The pre-SDD period ran from June 1, 2021, to June 30, 2022, and the post-SDD period ran from July 1 (start of intervention) to July 31, 2023.

Eligible patients for SDD application were mechanically ventilated (either on ICU admission or during ICU admission) and expected to remain ventilated until at least the second day after enrollment. Patients who were not initially expected to require 2 days of ventilation were rescreened and enrolled if the eligibility criteria were subsequently met.

Demographic variables were collected from all patients, ICU admission diagnosis, APACHE score (a severity of illness score ranging from 0 to 71 [APACHEII] [8], with higher scores indicating an increased risk of death, and specific risk factors for infection, including prior receipt of intravenous antibiotics. Other recorded data included the duration of mechanical ventilation, ICU and hospital length of hospital stay, and mortality.

### Infection surveillance

Infections were defined according to the ENVIN registry manual [13]:

Ventilation-associated pneumonia (VAP) was defined as pneumonia that develops in a patient on mechanical ventilation after intubation. The definition of VAP is based on an imaging test (chest X-ray or CT) and at least two of the following clinical-analytical criteria: fever >38° C with no other origin, leukopenia (<4,000 mm^3^) or leukocytosis (≥12. 000 mm^3^), appearance of purulent sputum or change in its characteristics, cough or dyspnea or tachypnea, suggestive auscultation (crackles, rhonchi, wheezing), impaired gas exchange (O2 desaturation or increased oxygen or ventilatory demands) and a positive microbiological test. Quantitative culture of the endotracheal aspirate specimen with was performed with a cutoff of 106 CFU/ml. Other samples would be bronchoalveolar lavage (BAL) with a cutoff of ≥ 104 CFU/ml or ≥ 5% of cells containing intracellular bacteria on direct microscopic examination, protected brush with a cutoff of ≥ 104 CFU/ml. Other positive microbiological findings to consider the diagnosis would be: positive blood culture unrelated to another focus of infection, positive growth in pleural fluid culture, positive pleural or lung abscess aspiration puncture, evidence of pneumonia on lung histological examination, or positive diagnosis of pneumonia due to viruses or particular microorganisms (Legionella, Aspergillus, Mycobacteria, Mycoplasma, Pneumocystis jirovecii)

Catheter-associated bloodstream infection (CLABSI) was defined as “Bacteremia or fungemia in a patient with a vascular device with one or more positive peripheral blood cultures, with clinical manifestations of infection (fever, chills, and/or hypotension), and without another apparent source of bloodstream infection”. In addition, at least one of the following conditions must be met: 1. Positive culture from the end of the catheter (15 colony forming units -CFU- by the semiquantitative method or 100 CFU from the quantitative culture) with identification of the same microorganism as in the blood (same species and antibiogram). 2. Simultaneous quantitative blood cultures were performed through the catheter and by venipuncture at a ratio of 4:1 (catheter blood vs. peripheral blood). 3. Differential time until bacterial growth is detected, of at least 2 h between the blood culture obtained by catheter and the peripheral blood culture, which is assurable only in laboratories with automated blood culture systems.

Primary bacteremia was defined as the sum of bacteremias of unknown origin and catheter-related bacteremias.

Secondary bacteremia is defined as the isolation of one or more micin isolated in one or more blood cultures in a patient with a known focus of infection.

Catheter-associated urinary tract infection (CAUTI) was defined as a urinary tract infection which the positive culture was obtained from an indwelling urinary catheter that was in place for more than 2 days.

In addition, patients with other community-acquired or nosocomial infections outside the ICU or in patients from other centers or hospitals were recorded.

As part of the clinical practice of our unit and following the recommendations of the Rzero program of the Spanish Society of Intensive Care Medicine and Coronary Units (SEMICYUC), routine microbiological surveillance is performed to detect colonization status. Samples of the pharyngeal and perianal exudates were taken upon on admission from all patients with SDD criteria and weekly from all patients during their stay in the unit.

### Intervention

Non-absorbable antibiotics were prepared by the Hospital Pharmacy Service following the rules for the right preparation and quality control of pharmaceutical formulations (Real Decreto 175/2001, 23 February) [14]. Both formulations (paste and liquid) had the same composition; thus, for each 125 mg (paste) or 125 ml (liquid) there were: 2.5 grams of colistin sulfate (Laboratorium Ofichem, Netherlands) plus 4 grams of gentamicin sulfate (Fujlan Fukang Pharmaceuticals Co. Ltd, China) plus 2.5 grams of nystatin (SC Antibiotice, Romania). The protocol was applied to the selected patients after adequate oral hygiene using 0.1% aqueous chlorhexidine solution and aspiration of secretions. It consisted of: 1) topical application into the buccal mucosa and oropharynx of 0.5 grams of paste every six hours; 2) application of 20 milliliters of SDD liquid every six hours via nasogastric tube (NGT) or orally if the patient did not have an NGT; 3) administration of an intravenous third-generation cephalosporin (cefotaxime, Normon Laboratories, Spain), 1 gram every 6 hours, during the first four days of ICU admission unless already treated with specific antibiotics with activity against Gram-negative bacteria, in which case no additional antibiotics were administered.

All other treatments, including the use of prophylactic or therapeutic antibiotics, were at the discretion of the treating clinicians, ins in accordance with respective institutional microbiological prescription policies.

### Outcomes

The primary outcome was all-type nosocomial ICU infection during the index ICU admission. The secondary outcomes were ventilation-associated pneumonia (VAP), catheter-associated bloodstream infection (CLABSI), and secondary bacteriemia and catheter-associated urinary tract infection (CAUTI). Microbiological secondary outcomes were the incidence of new pre-defined antibiotic-resistant organisms (MDRO) from all blood, non-blood surveillance, and clinical cultures. For epidemiologic purposes, MDROs are defined as microorganisms, predominantly bacteria, that are resistant to one or more classes of antimicrobial agents. Although the names of certain MDROs describe resistance to only one agent (e.g., MRSA, VRE), these highly resistant organisms deserve special attention in healthcare facilities. Pharmacological outcomes were total antibiotic use, defined in daily defined doses.

Incidence density was calculated as the percentage of patients out of the total who developed an infection, were colonized, or had per thousand days of use in the case of a device-associated infection.

### Ethical considerations

This study was approved by the Ethics Committee of biomedical research ethics committee, in which our Hospital is included, with reference n° 0600-N-23. In addition, the ENVIN registry is authorized by the Ministry of Health. The Ethics Committee allowed the waiver of informed consent from patients or their relatives of those who did not receive the SDD. This was obtained from those who were administered the SDD. The data, aggregated and anonymized, were processed in accordance with national and European regulations on personal data protection.

### Statistical analysis

The results are expressed descriptively as means, medians, and proportions, according to the type of variable. Incidence densities were calculated and compared between groups using Epidat 4.2 software [15]. Univariate analysis was performed using Student’s t test for independent samples. Considering the variable “all infections” as the outcome multivariate analysis using the stepwise logistic regression test was used with variables statistically significant in the univariate and those considered clinically relevant. A p<0.05 was considered significant for all analysis.

## Results

**Fig. 1.**
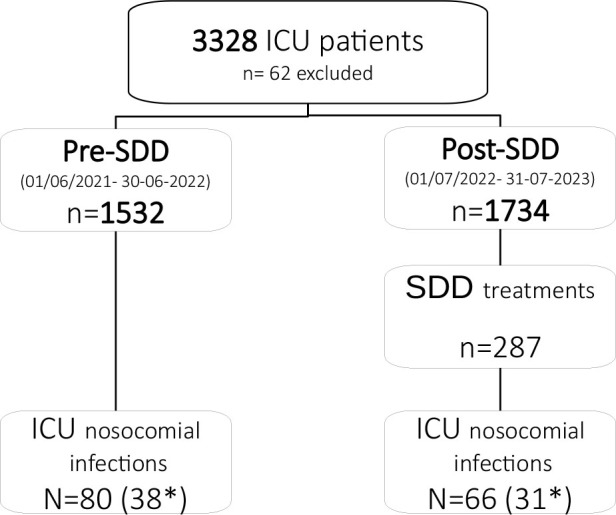
Study flowchart. SDD: Selective digestive decontamination. ICU: Intensive care unit. * ICU nosocomial infection ENVIN type

During the study, 3.328 patients were admitted in the ICU. Sixty-two patients were excluded because they died within 24 hours of admission, 26 in the first period and 36 in the second period. As a result, the pre- and post-SDD cohorts comprise 1532 and 1734 patients each. (Figure 1. Patient flow).

The baseline characteristics of the patient population, including infections before ICU admission, are shown in Table 1. The severity of the patients was similar in both periods, as was the mean age and sex. The main reasons for admission were medical and cardiac diagnosis. A large number of cardiac patients is noteworthy, given that our unit has 8 beds available for this type of patient. These patients have less APACHE and shorter length of stay. In the second period, admissions from the community were slightly higher. Hospital stay, ICU stay and mortality were similar.

**Table 1.** Baseline clinical characteristics of patients.

	**Pre SDD (n=1532)**	**Post SDD (n=1734)**	**p**
Sex (M/F) (%)	67.4/32.6	65.3/34.7	0.38
Age (years); (Mean ± SD)	61.5 ± 16.1	63.1 ± 15.5	0.135
Diabetes (%)	25.8	25.4	0.49
Chronic kidney failure (%)	11.4	11.3	0.5
Chronic obstructive pulmonary disease (%)	11.8	11.5	0.48
Cirrhosis (%)	3.5	3.9	0.41
Inmunocompromised (%)	8.7	8.9	0.46
APACHE II; (Mean ± SD)	14.7 ± 9.4	15.7 ± 9.8	0.44
SOFA admission; (Mean ± SD)	6.7 ± 3.2	6.3 ± 4.1	0.81
Admission Diagnosis (%)			
Cardiological	41.3	40.5	
Medical	38.9	41.5	0.75
Surgical	16.1	14.3	
Trauma	3.7	3.7	
Septic Shock (%)	5.2	6.1	0.08
Use of vasoactive medication (%)	42.1	43.7	0.35
Non-invasive MV (%)	8.3	8.7	0.40
Invasive MV (%)	20.4	19.5	0.30
Enteral or parenteral nutritional support (%)	25.2	23.4	0.42
Renal replacement therapies (%)	4.1	4.6	0.33
Communitary infection (%)	18.5	24.6	0.021*
Nosocomial ICU infection (%)	4.5	3.8	0.16
Nosocomial out of ICU infection (%)	6.1	6.0	0.95
Nosocomial infection from another hospital (%)	1.4	1.2	0.50
Length of MV (median, IQR)	4.1 (2 – 8)	4.5 (2 – 9)	0.45
Length of ICU stay (median, IQR)	4.5 ± 7.6	4.1 ± 6.3	0.28
Mortality rate (%)	10.4	12.6	0.08

SDD: Selective digestive decontamination; APACHE: Acute physiology and chronic health evaluation; SOFA: Sequential organ failure assessment; ICU: Intensive care unit; SD: standard deviation; IQR: Interquartile range; MV: Mechanical ventilation

The percentage of patients receiving ventilator support was similar in the two periods. In the first one, 20.38% (n= 312) and in the second one, 19.46% (n= 337). In the second period, the number of patients requiring SDD treatment, i.e. those who had been on mechanical ventilation for more than 24 hours) was 287.

**Fig. 2.**
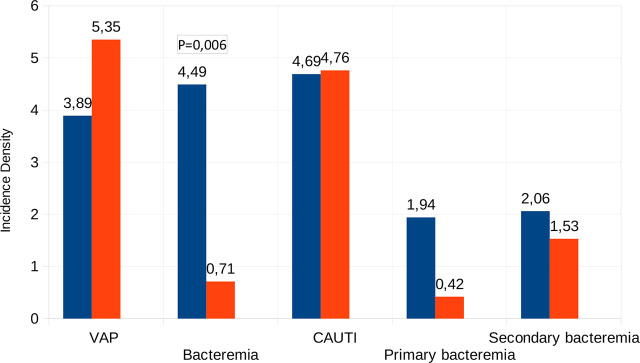
**Distribution of device-associated infection in both periods.** VAP: ventilator associated pneumonia. Bacteremia is the sum of primary and catheter line associated bloodstream infection (CLABSI). Incidence density is expressed as number per thousand days of use of device, except fo secondary bacteremia (per days of ICU stay)

The difference between the two periods in the incidence of total ICU nosocomial infections and device-associated infections is shown in Table 2. Overall, there were fewer infections in the second period (6,54 vs 9,21), without reaching statistical significance, being the risk ratio for all infections 0,711 (0,428–1,172), p=0,16. Considering each type of infection separately, there was a significant decrease in primary and catheter associated bacteremias (p=0,006). The CAUTI has not changed in both periods and, with respect to VAP, has decreased slightly. Figure 2 shows the distribution of device-associated infections in both periods. As there was no variable statistically significant in the univariate analysis between periods, we added all relevant clinical variables in the multivariate alongside the SDD period, but none were included in the model.

**Table 2.** ENVIN type infections

**Infections**	**Pre-SDD (n=1532)**	**Post-SDD (n=1734)**
Nosocomial infection ENVIN type (n)	38	31
Ventilator-associated pneumonia (VAP)	6	8
Bacteremia (primary and CLABSI)	11	2
Primary bacteremia	3	2
Secondary bacteremia	6	4
Catheter associated urinary tract infection (CAUTI)	15	17

SDD: Selective digestive decontamination; CLABSI: Catheter line associated bloodstream infection

The results related to colonization and MDROs found in both periods are shown in Table 3. Colonization reduction focused on colonization acquired during ICU stay, with similar admission rates between the two periods. The most striking result was a decrease in the occurrence of carbapenemase-producing Enterobacterales (CPE) in the post-SDD period compared to pre-SDD period. The detection of CPE bacteria decreased from 33 to only 7, resulting in a significant decrease.

**Table 3.** Colonization incidence density and MDRO between periods

	**Post-SDD**	**Pre-SDD**	**RR (CI95%)**	**p**
Colonization ID	2,36	3,26	0.724 (0.467–1.117)	0.127
Colonization at admission ID	1,73	1,95	0.884 (0.515–1.517)	0.633
Colonization during ICU stay ID	0,63	1,31	0.486 (0.210–1.064)	0.053
Surveillance samples (n)	1645	1831		
Positive samples (n)	54	161		
Colonized patients (n)	41	50		
MDRO (n)	43	67		
Positive samples (%)	3,28	8,79	0.37 (0.25–7.08)	<0.0001
Colonized patients (%)	2,67	2,88	0.92 (0.75–1.13)	0.7064
MDRO (%)	2,41	3,32	0.71 (0.45–8.3)	0.07
MARSA (n)	2	2		
Betalactamases (ESBLs) (n)	31	23		
Carbapenemases (n)	7	37		
Pseudomonas MDR	2	3		
Acinetobacter spp MDR	3	0		

ID: Incidence density (per 100 patients); SDD: Selective digestive decontamination; ICU: Intensive care unit; MDRO: drug-resistant organisms; MRSA Methicillin-resistant Staphylococcus aureus; ESBLs: extended-spectrum betalactamases; RR: risk ratio; CI: confidence interval

## Discussion

The main finding of this study was the significant reduction in the incidence of bacteremia following the implementation of the use of SDD in our ICU. We have not found a decrease in the rest of the infections studied, including pneumonias associated with mechanical ventilation. This finding is relevant as it is the first time it has been reported in an ICU with a low rate of infections and MDRO. We believe that the effect may be due to control of intestinal bacterial overgrowth and decreased colonization of the patient’s skin or caregivers’ hands since the decrease has focused on bacteremia related to intravenous catheters. Reduced bacterial translocation may also have played a role, albeit to a lesser extent.

After the COVID pandemic and because of the increase in the rates of nosocomial infections and incidence of MDRO occurring at national level [16] and in our own ICU, we decided to implement the SDD recommendation of Zero-Pneumonia Project, not yet incorporated into the bundle of measures used to prevent VAP. However, in the period studied, the incidence of both NI and MDRO returned to a low-rate situation, similar to pre-pandemic, so the impact of SDD turned out to be lower than anticipated.

Regarding the impact of SDD on the incidence of bacteremia, the literature reports mixed and conflicting results. Of the two most important systematic reviews [7,17], one found a decrease in bacteremias in general (RR: 0.68 [95% CI: 0.57–0.81]) and the other only in the group of non-catheter-associated bacteremias caused by Gram negative bacteria (OR: 0.39 [95% CI: 0.24–0.63]). In the latter study, they observed that in the group of catheter-associated bacteremias and in the group of non-catheter-associated bacteremias caused by gram-positive microorganisms, SDD showed no significant effect. More recently, Wittekamp et al. in a cluster clinical trial, also reported the failure of a SDD (albeit without parenteral antibiotics) in the prevention of bacteremia [18].

The lack of effect of SDD on intravascular catheter-associated bacteremia is based on the hypothesis that this type of infection is predominantly exogenous and that it can be reduced with exclusive hygienic measures. The fact that the bacteremias that have been most reduced in our study are those associated with catheters requires an explanation, as these bacteremias are considered to be caused by the passage of microorganisms from the skin or hands of the caregivers into the bloodstream via the devices carried by the patient. Our hypothesis is that by decreasing the intestinal bacterial load, the subsequent dissemination that can occur through the hands of caregivers from the perianal area to the skin of patients and invasive devices that are handled by them is reduced, thereby preventing exogenous infections. This would be a similar mechanism that justifies the use of daily chlorhexidine gluconate (CHG) baths, using prepackaged cloths. This is a well-supported evidence-based practice to reduce patient’s risk of acquiring a central line associated bloodstream infection (CLABSI) [19]. Although we cannot rule out that the effect was due to other factors, as this was not a concurrent study but a quasi-experimental study, we do not consider this likely as no other preventive measures were introduced during the postintervention period, nor were any training or hygiene control activities or changes in the type of intravenous catheters used.

In our study, we did not find any effect on the prevention of VAP. This has been the most consistent effect of this therapy, calculating that for approximately 5 patients treated, one respiratory infection is prevented [8]. These results have recently been corroborated in a systematic review and meta-analysis of 22 studies involving 3,619 patients, in which the use of SDD was associated with a 56% reduction in the risk of VAP [7]. However, the level of evidence is considered very low due to the biases associated with the different studies considered. In this sense, the main source of bias is related to the criteria used for diagnosis. In our study, the assignment is made by a small number of researchers, who have been homogeneously trained in the ENVIN study criteria, so we believe that it has been minimized.

We have also not been able to demonstrate an effect on in-hospital mortality. Obviously, this was not within our objectives due to the sample size and mortality rate in our ICU. We did not include the type of patients for whom the impact of SDD has been most demonstrated, namely patients with acute brain injuries [20]. To determine whether SDD has a positive result on a hard clinical outcome, large studies with rigorously selected predefined patient subgroups will be needed [21].

Finally, related to the safety of SDD, we found a reduction (though not statistically significant) in the rate of multidrug-resistant organisms. This result is in line with the numerous studies of all types (ecological, follow-up cohorts and meta-analyses) that have shown that the use of SDD is not associated with an increase in antibiotic resistance [22,23,6,7]. In fact, several of them report a decrease either in general, in groups of bacteria (gram-positive or negative) or in the microbiota with specific resistance to certain antimicrobials [24,25]. In our study, the significance was related to the marked decrease in carbapenem-resistant Enterobacterales. In most published studies, the greatest reduction is found in Enterobacterales with extended-spectrum betalactamases (ESBL) [26], but other multidrug-resistant bacteria (Pseudomonas, Acinetobacter) and, as the studies become more recent, with the presence of carbapenemases are also found. In fact, SDD is being used successfully in the eradication of the carrier state of this type of resistance [27]. In our case, the use of cefotaxime as the intravenous antibiotic was followed by a nonsignificant increase in Enterobacterales with extended-spectrum beta-lactamases (ESBL) without any other apparent side effects.

One of the main concerns regarding the use of SDD is its medium- to long-term impact on both the ecology of ICUs and the microbiota of the patients. In our case, we have only monitored the colonization and infection of patients during their stay in the ICU. However, there are several studies that analyze longer periods of time [26]. Two of them, in ICUs in our geographical and organizational environment [28,29] carried out in medical-surgical ICUs and with a follow-up of 5 and 4 years respectively, reported that the incidence of carriers of resistant pathogens remained stable. In the first study, the incidence of Enterobacterales resistant to antimicrobials in the SDD paste did not change and Pseudomonas aeruginosa resistance to tobramycin and amikacin was significantly reduced. In the second study, despite increasing rates of Colistin- and tobramycin-acquired ICU colonization resistance by 1000 days, if adjusted by the rate of resistances at admission, were non-significant. In the longest cohort study [30], with a SDD use period of 21 years, the rates of ICU-acquired resistant organisms did not increase significantly over time, even though baseline rates of resistant strains measured at admission increased over time. In view of these results and new evidence recently provided, it can be concluded that prolonged use of SDD does not translate into a negative impact on the ecology of antimicrobial resistance.

Strengths and limitations: Despite being a prospective study, the limited sample size precludes conclusive results on the relationships between SDD and the mechanisms involved in infection control. The fact that the study was conducted in an ICU with a large percentage of short-stay, complex patients (acute cardiac) resulted in relatively low infection rates, thus reducing the effect of treatment. We have also not been able to extend the effect to other significant clinical outcomes, such as mortality. However, we believe that it is necessary to determine the effect of this treatment as a form of infection control in ICUs in all possible scenarios.

## Conclusion

In an ICU with a low rate of nosocomial infections and MDRO, implementation of an SDD protocol reduced the incidence of catheter-associated bacteremia and colonization by this type of bacteria.
